# Two new *Lesteva* Latreille (Coleoptera, Staphylinidae, Omaliinae) from Longwangshan Mountain, East China

**DOI:** 10.3897/zookeys.194.3139

**Published:** 2012-05-17

**Authors:** Wen-Li Ma, Li-Zhen Li, Mei-Jun Zhao

**Affiliations:** 1 Department of Biology, College of Life and Environmental Sciences, Shanghai Normal University, Shanghai, 200234, P. R. China

**Keywords:** Coleoptera, Staphylinidae, *Lesteva*, new species, key, Longwangshan, East China

## Abstract

Two new species of *Lesteva* Latreille are described and illustrated from Longwangshan Mountain, East China, *viz*. *Lesteva cala* Ma & Li, **sp. n.** and *Lesteva erythra* Ma & Li, **sp. n.** The latest key to Chinese *Lesteva* is modified to include the species described from continental China since 2000.

## Introduction

*Lesteva* Latreille is one of the largest omaliine genera of the family Staphylinidae with over 100 species worldwide, among which 18 are known from China (Cameron 1934, Rougemont 2000, Watanabe 2005, Li et al. 2005). So far, eight species have been recorded from Longwangshan mountain, Zhejiang Province, East China (Rougemont 2000 (7 spp.), Li et al. 2005 (1 sp.)).

In April 2004, the staff of the junior author’s laboratory surveyed the staphylinid fauna of Longwangshan Mountain, during which a large series of *Lesteva* were collected. On closer examination these beetles were found to belong to two new species. Since there are three hitherto known subgenera of the genus: *Lesteva* Latreille, 1797, *Lestevidia* Jeannel & Jarrige, 1949 and *Lestevina* Bordoni, 1999, we arrange both new species into the subgenus *Lesteva* Latreille based on the definition of subgenera. In the present paper, the new species are described and illustrated, with modified couplets of the latest key (Rougemont 2000) to Chinese *Lesteva*.

## Material and methods

All measurements are in millimeters. The following abbreviations are used in the text:

**BL** – length of the body from the labral anterior margin to the anal end; **FL** – length of the body from the labral anterior margin to the elytral apex; **HL** –length of the head from the clypeal anterior margin to the head base; **HW** –maximum width of the head; **PL** –length of the pronotum along the midline; **PW** –maximum width of the pronotum; **EL** –length of the elytra from the apex of the scutellum to the elytral posterior margin; **EW** – maximum width of the elytra.

The holo- and paratypes are deposited in the Insect Collection of Shanghai Normal University, Shanghai, China (**SNUC**).

## Descriptions

### Lesteva (Lesteva) cala

Ma & Li sp. n.

urn:lsid:zoobank.org:act:4F0B6E1B-ECA0-49AB-92DD-5E6377941C98

http://species-id.net/wiki/Lesteva_cala

Figs 1 3–5

#### Type locality. 

Longwangshan Mountain, East China

#### Type material

(38 ♂♂, 35 ♀♀)**. Holotype:** **CHINA: Zhejiang Prov.:** ♂, Anji County, Mt. Longwangshan, 25.iv.2004, alt. 950–1,200 m, Liang Tang leg. **Paratypes:** **CHINA:Zhejiang Prov.,** **Anji County,** **Mt. Longwangshan:** 5 ♂♂, 3 ♀♀, same label data as holotype; 3 ♀♀, 25.iv.2004, alt. 950–1,200 m, Jing-Wen Zhu leg.; 1 ♀, 25.iv.2004, alt. 950–1,200 m, Jia-Yao Hu leg.; 1 ♀, 25.iv.2004, alt. 950–1,200 m, Jing Chen leg.; 2 ♀♀, 25.iv.2004, alt. 950–1,200 m, Li-Long Zhu leg.; 2 ♂♂, 2 ♀♀, 24. iv.2004, alt. 300–500 m, Jing-Wen Zhu leg.; 2 ♂♂, 24.iv.2004, alt. 300–500 m, Jia-Yao Hu leg.; 3 ♂♂, 2 ♀♀, 24.iv.2004, alt. 300–500 m, Jing Chen leg.; 5 ♂♂, 24.iv.2004, alt. 300–500 m, Xin-Jin Li leg.; 8 ♂♂, 4 ♀♀, 24.iv.2004, alt. 300–500 m, Liang Tang leg.; 3 ♀♀, 24.iv.2004, alt. 300–500 m, Huang & Chi leg.; 1 ♂, 3 ♀♀, 24.iv.2004, alt. 300–500 m, Li-Long Zhu leg.; 4 ♂♂, 5 ♀♀, 23.iv.2004, alt. 300–500 m, Li-Long Zhu leg.; 3 ♂♂, 2 ♀♀, 23.iv.2004, alt. 300–500 m, Jing-Wen Zhu leg.; 2 ♂♂, 4 ♀♀, 23.iv.2004, alt. 300–500 m, Liang Tang leg.; 1 ♂, 23.iv.2004, alt. 300–500 m, Jing Chen leg.; 1♂, 23.iv.2004, alt. 300–500 m, Li & Hu leg.

#### Description.

Measurements and ratios: BL 3.3–3.9. Holotype: HL 0.59, HW 0.61, PL 0.63, PW 0.67, EL 1.24, EW 1.20, HL/HW 0.97, HW/PW 0.91, PL/PW0.94.

**Figures 1–2.**
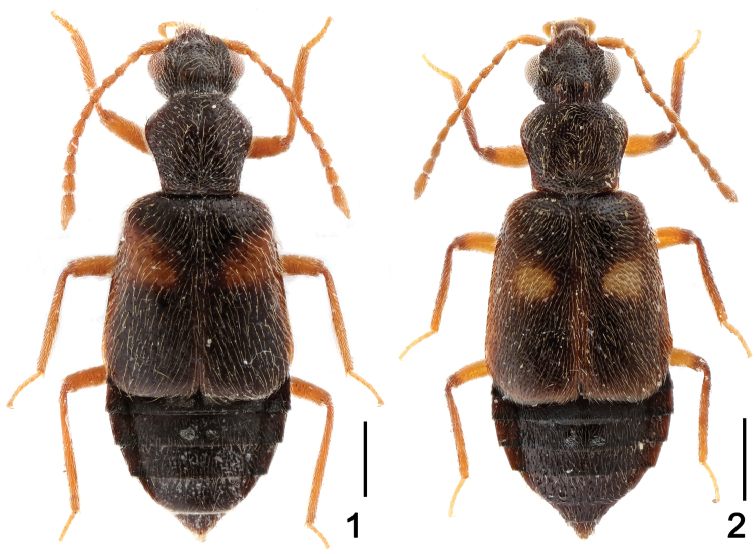
Male habitus of *Lesteva* **1** *Lesteva cala* **2** *Lesteva erythra*. Scales: 0.5 mm.

Habitus as in Fig. 1. Black, mouth-parts, antennae and legs uniformly rufo-testaceous. Each elytron with a dark red subhumeral macula. Pubescence pale, evident and recumbent on whole body.

Head sub-triangular, coarsely and sparsely punctate, widest across eyes and slightly broader across compound eyes than long; vertex moderately depressed; eyes prominent; ocelli distinct, distance between them slightly larger than that from lateral margin of each ocellus to mesal margin of eye; postocular margins about half length of longitudinal diameter of eye; antennae moderately long, overlapping base of pronotum by two terminal segments when pulled posteriad; antennomeres I broad at middle, antennomeres II much shorter and narrower than I, antennomeres III–XI gradually thickened; relative length of each antennomere from base to apex as 10 : 6 : 7.5 : 6.5 : 5.5 : 5.5 : 6 : 5.5 : 6.5 : 6 : 12.

Pronotum subcordate, moderately convex, widest near anterior third, slightly wider than head; lateral margins arcuate at anterior two-thirds and nearly straight at posterior third; punctation and pubescence similar to those on head; disc with shallow U-shaped depressed area. Scutellum subtriangular, surface with fine punctation and pubescence.

Elytra subtrapezoidal, gradually dilated posteriorly, posterior angles broadly rounded; punctation and pubescence distinctly finer and sparser than those on pronotum.

Abdomen broad, widest at segment IV (first visible abdominal segment), then distinctly narrowed posteriorly. Tergites with dense, fine punctation and decumbent pubescence; tergites IV–V each provided with one pair of tomentose admesal patches.

**Figures 3–8.**
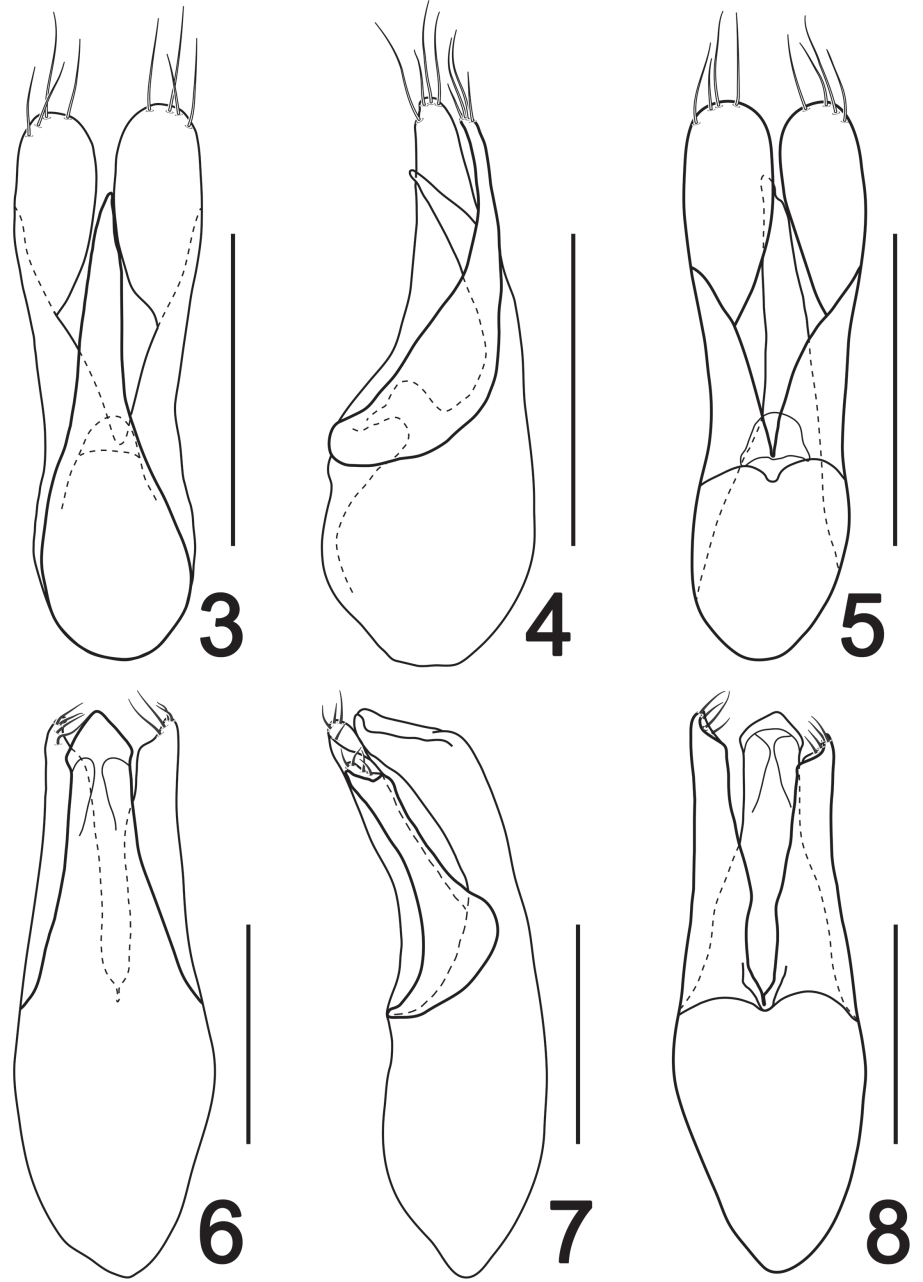
Aedeagus of *Lesteva* (**3, 6** dorsal view **4, 7** lateral view **5, 8** ventral view). **3–5** *Lesteva cala* **6–8**  *Lesteva erythra*. Scales: 0.2 mm.

Male. Sternite VIII transverse, posterior margin broadly emarginate. Aedeagus (Figs 3–5) length 0.33 mm; median lobe narrow and much shorter than parameres, gradually narrowed apically; parameres symmetrical, each distinctly roundly broadened in apical half, with four long apical setae.

Female. Protarsomeres I–IV not dilated. Otherwise similar to male.

#### Distribution.

East China: Zhejiang Province.

#### Bionomics.

The type series were obtained by sifting leaf litter and wet moss at the streamside of the Longwangshan Mountain in late April.

#### Etymology.

The specific name ‘*cala*’ is a latinized Greek adjective and means‘beautiful’.

#### Remarks.

The new species is close to *Lesteva huabeiensis* Rougemont from Beijing in sharing similar elytral maculae, but it can be readily separated by the aedeagus with parameres much longer than the median lobe, *Lesteva huabeiensis* has the aedeagus with the median lobe slightly shorter than the parameres. *Lesteva cala* also resembles *Lesteva pulcherrima* Rougemont in the similar form of parameres, but differs in having a much narrower median lobe of the aedeagus and dark coloration of the body.

### Lesteva (Lesteva) erythra

Ma & Li sp. n.

urn:lsid:zoobank.org:act:22C2925D-7A2A-4FCA-8B04-2FB0D208CA4F

http://species-id.net/wiki/Lesteva_erythra

Figs 2 6–8

#### Type locality.

Longwangshan Mountain, East China

#### Type material

(44 ♂, 36 ♀)**. Holotype:** **CHINA: Zhejiang Prov.:** ♂, Anji County, Mt. Longwangshan, 24.iv.2004, alt. 300–500 m, Xin-Jin Li leg. **Paratypes: CHINA: Zhejiang Prov.,** **Anji County ,** **Mt. Longwangshan:** 10 ♂♂, 8 ♀♀, 25.iv.2004, alt. 950–1,200 m, Jing-Wen Zhu leg.; 7 ♂♂, 4 ♀♀, 25.iv.2004, alt. 950–1,200 m, Liang Tang leg.; 5 ♂♂, 5 ♀♀, 25.iv.2004, alt. 950–1,200m, Li-Long Zhu leg.; 2 ♀♀, 25.iv.2004, alt. 950–1,200 m, Xin-Jin Li leg.; 1 ♂, 3 ♀♀, 25.iv.2004, alt. 950–1,200 m, Jing Chen leg.; 11 ♂♂, 4 ♀♀, 24.iv.2004, alt. 300–500 m, Jing-Wen Zhu leg.; 1 ♀, 24.iv.2004, alt. 300–500m, Liang Tang leg.; 1 ♀, 24.iv.2004, alt. 300–500 m, Jia-Yao Hu leg.; 1 ♀, 24.iv.2004, alt. 300–500 m, Jing Chen leg.; 3 ♀♀, 24.iv.2004, alt. 300–500 m, Li-Long Zhu leg.; 7 ♂♂, 1 ♀, 23.iv.2004, alt. 300–500 m, Jing-Wen Zhu leg.; 1 ♂, 23.iv.2004, alt. 300–500 m, Li-Long Zhu leg.;1 ♂, 3 ♀♀, 23.iv.2004, alt. 300–500 m, Liang Tang leg.

#### Description.

Measurements and ratios: BL 2.8–3.5. Holotype: HL 0.54, HW 0.52, PL 0.56, PW 0.54, EL 1.24, EW 1.17, HL/HW 1.04, HW/PW 0.93, PL/PW 1.04.

Habitus as in Fig. 2. Black, elytra usually paler, darkish brown, suture and lateral margins reddish; mouth-parts fuscous brown; antennae and legs reddish brown, except for apex of femora and large part of tibia which are infuscate; each elytron with one small round orange-yellow spot near middle. Pubescence pale, evident and recumbent on whole body.

Head subtriangular, widest across eyes and slightly broader across compound eyes than long, densely and coarsely punctate, vertex moderately depressed; eyes prominent; ocelli distinct, distance between them equal to that from lateral margin of each ocellus to mesal margin of eye; postocular margins less than half length of longitudinal diameter of eye; antennae elongate, extending beyond posterior margin of pronotum by three segments when pulled posteriad, antennomeres I moderately robust, antennomere II much shorter and narrower than I, antennomeres III–XI gradually thickened; relative length of each antennomere from base to apex as 9 : 5.5 : 6 : 5.5 : 6 : 6 : 5 : 5.5 : 5.5 : 5 : 10.

Pronotum subcordate, slightly convex, widest near anterior third, slightly wider than head; lateral margins arcuate at anterior two-thirds and nearly straight at posterior third; more coarsely and densely punctate than on head, with slightly deep U-shaped depression on disc. Scutellum subtriangular, scattered with fine punctation and pubescence.

Elytra subtrapezoidal and rather flat, gradually dilated posteriorly, posterior angles broadly rounded, coarsely and sparsely punctate, covered with fine pubescence as on pronotum.

Abdomen broad, widest at segment IV (first visible abdominal segment), then distinctly narrowed posteriorly. Tergites with dense, fine punctation and decumbent pubescence; tergites IV–V each with one pair of tomentose admesal patches.

Male. Sternite VIII transverse, with apical margin distinctly emarginate. Aedeagus (Figs 6–8) length 0.50 mm; median lobe moderately broad, gradually narrowed apically; parameres slightly asymmetrical, subequal in length as median lobe, with four short apical setae.

Female. Protarsomeres I–IV not dilated. Otherwise similar to male.

#### Distribution.

East China: Zhejiang Province.

#### Bionomics.

All the type specimens were obtained from the same habitat as the preceding species.

#### Etymology.

The specific epithet ‘*erythra*’ means ‘reddish’, referring to the body color of the new species.

#### Remarks.

The new species is placed close to *Lesteva flavopunctata* Rougemont in sharing a similar form of the elytral spots, but can be readily separated by the body being somewhat spindle-shaped. *Lesteva erythra* also resembles *Lesteva flavopunctata* in having a similar aedeagal form, but differs by the aedeagus with the parameres subequal in length as median lobe, while *Lesteva flavopunctata* has the aedeagus with the parameres shorter than the median lobe.

### Modified couplets of Rougemont’s key

**Note.** Rougemont’s key (Rougemont 2000) for the identification of Chinese *Lesteva* species is modified as follows to include the taxa described from continental China since 2000.

**Table:** 

19(20)	Elytra each with a well or poorly defined oblique pale macula; legs entirely testaceous	21
20(19)	Elytra each with a round spot situated near middle; body fuscous, deep black, or metallic blue; at least tibia more or less infuscate	27
21(22)	Pronotum and elytra (excluding maculae) bicolorous	23a
22(21)	Pronotum and elytra (excluding maculae) concolorous, black	25a
23a(23b)	Elytra subtrapezoidal; Elytral maculae situated in anterior half of elytra	23
23b(23a)	Elytral somewhat parallel-sided; Elytral maculae extending from humeral angles to 1/3 of suture from posterior margin	*Lesteva ochra* Li, 2005
23(24)	Head and pronotum brown or pitchy; elytra testaceous, maculae pale testaceous. Outline of fore-body: Fig. 4; aedeagus: Fig. 17	*Lesteva huabeiensis* Rougemont, 2000
24 (23)	Head and pronotum black; elytra dark brown, maculae reddish; male unknown	sp. C
25a(25b)	Smaller(3.3–3.9mm); elytra about as broad as their length; surface of pronotum with a distinct U-shaped depression. Outline of fore-body: Fig. 1; aedeagus: Fig. 3–5.	*Lesteva cala* Ma & Li, sp. n.
25b(25a)	Larger(3.9–4.3mm); elytra distinctly elongate; surface of pronotum devoid of clear impressions	25
25(26)	Elytral maculae smaller, obscure, dark red; puncturation of pronotum and elytra coarse, deep, interstices narrower than diameter of punctures. Outline of fore-body: Fig. 5; aedeagus: Fig. 23a, b, c	*Lesteva submaculata* Rougemont, 2000
26 (25)	Elytral maculae large, bright orange, clearly defined and occupying most of anterior half of elytra; puncturation of pronotum deep but sparse, interstices larger than diameter of punctures; puncturation of elytra fine, sparse and shallow, surface of whole fore-body shiny. Outline of fore-body: Fig. 6; aedeagus: Fig. 21a, b.	*Lesteva elegantula* Rougemont, 2000
27(28)	Femora testaceous, tibiae more or less infuscate; each elytron with a round orange spot; smaller species (≤3.5mm).	27a
28(27)	Femora and tibiae entirely black , or with a blue metallic reflex; larger species (4.5–5.0mm).	29
27a(27b)	Body parallel-sided; parameres shorter than median lobe of the aedeagus	*Lesteva flavopunctata* Rougemont, 2000
27b(27a)	Body somewhat spindle-shaped; parameres almost as long as median lobe of the aedeagus. Outline of fore-body: Fig. 2; aedeagus: Fig. 6–8.	*Lesteva erythra* Ma & Li, sp. n.

## Supplementary Material

XML Treatment for Lesteva (Lesteva) cala

XML Treatment for Lesteva (Lesteva) erythra
